# Demonstration and Performance Evaluation of Two Novel Algorithms for Removing Artifacts From Automated Intraoperative Temperature Data Sets: Multicenter, Observational, Retrospective Study

**DOI:** 10.2196/37174

**Published:** 2022-10-05

**Authors:** Amit Bardia, Ranjit Deshpande, George Michel, David Yanez, Feng Dai, Nathan L Pace, Kevin Schuster, Michael R Mathis, Sachin Kheterpal, Robert B Schonberger

**Affiliations:** 1 Department of Anesthesiology Critical Care and Pain Medicine Massachusetts General Hospital Boston, MA United States; 2 Department of Anesthesiology Yale School of Medicine New Haven, CT United States; 3 Department of Anesthesiology University of Utah Salt Lake City, UT United States; 4 Department of Surgery Yale School of Medicine New Haven, CT United States; 5 Department of Anesthesiology University of Michigan School of Medicine Ann Arbor, MI United States

**Keywords:** temperature, intraoperative, artifacts, algorithms, perioperative, surgery, temperature probe, artifact reduction, data acquisition, accuracy

## Abstract

**Background:**

The automated acquisition of intraoperative patient temperature data via temperature probes leads to the possibility of producing a number of artifacts related to probe positioning that may impact these probes’ utility for observational research.

**Objective:**

We sought to compare the performance of two de novo algorithms for filtering such artifacts.

**Methods:**

In this observational retrospective study, the intraoperative temperature data of adults who received general anesthesia for noncardiac surgery were extracted from the Multicenter Perioperative Outcomes Group registry. Two algorithms were developed and then compared to the reference standard—anesthesiologists’ manual artifact detection process. Algorithm 1 (a slope-based algorithm) was based on the linear curve fit of 3 adjacent temperature data points. Algorithm 2 (an interval-based algorithm) assessed for time gaps between contiguous temperature recordings. Sensitivity and specificity values for artifact detection were calculated for each algorithm, as were mean temperatures and areas under the curve for hypothermia (temperatures below 36 °C) for each patient, after artifact removal via each methodology.

**Results:**

A total of 27,683 temperature readings from 200 anesthetic records were analyzed. The overall agreement among the anesthesiologists was 92.1%. Both algorithms had high specificity but moderate sensitivity (specificity: 99.02% for algorithm 1 vs 99.54% for algorithm 2; sensitivity: 49.13% for algorithm 1 vs 37.72% for algorithm 2; F-score: 0.65 for algorithm 1 vs 0.55 for algorithm 2). The areas under the curve for time × hypothermic temperature and the mean temperatures recorded for each case after artifact removal were similar between the algorithms and the anesthesiologists.

**Conclusions:**

The tested algorithms provide an automated way to filter intraoperative temperature artifacts that closely approximates manual sorting by anesthesiologists. Our study provides evidence demonstrating the efficacy of highly generalizable artifact reduction algorithms that can be readily used by observational studies that rely on automated intraoperative data acquisition.

## Introduction

Body temperature is a critical vital sign, and its measurement during surgery is an integral part of standard American Society of Anesthesiologists monitoring [1,2]. Intraoperative hypothermia has been associated with perioperative complications, such as surgical wound infections, cardiac morbidity, coagulopathy, impaired drug metabolism, and prolonged recovery [3-7]. Given its profound impact on postoperative outcomes, accurately accounting for intraoperative temperature in large perioperative database studies is of paramount importance. Unfortunately, intraoperative temperature readings usually contain a number of artifacts. Mechanistically, these artifacts may be a result of temperature probes that are suboptimally placed, temperature probes that accidentally fall out of a patient’s oral cavity or nasal orifice, low readings resulting from the probe warming up from room temperature to a patient’s core temperature, or readings associated with the repositioning of temperature probes [8,9]. Although some studies have proposed temperature artifact–reducing algorithms, their validation remains lacking, and the most widely cited algorithm relies on equal time intervals across measurements—a condition that is frequently violated within many large data sets [10]. Our study aims to address these knowledge and performance gaps, as we compare the performance of two novel temperature artifact reduction algorithms to that of manual artifact removal by three board-certified anesthesiologists, using a large intraoperative temperature database.

## Methods

### Ethics Approval

This study was approved by the institutional review board (approval number: HIC 1206010438).

### Study Design

This was a multicenter, observational, retrospective study of data that were collected by the Multicenter Perioperative Outcomes Group (MPOG) consortium after institutional review board approval. The MPOG registry contains the anesthetic data of over 14 million procedures from over 48 medical centers. This consortium has rigorously collected and standardized information regarding anesthetic and surgical encounters with patient-level data [11]. The number of individual surgical procedures, the diversity of participants, and its wide geographic coverage make this database a very rich data source for drawing precise and reliable estimates. Both large academic medical centers and community hospitals contribute to this database, thereby yielding a large, representative, national sample. This database is among the largest anesthetic registries in the United States, and algorithm evaluation via the use of this registry would make algorithms generalizable across a wide array of institutions.

The study plan, including the sample size assessment, was published prior to data extraction and analysis [12].

### Inclusion and Exclusion Criteria

Anesthetic records of patients aged over 18 years who were undergoing general anesthesia with an endotracheal tube for noncardiac surgery were included in this study. The exclusion criteria comprised cases with an American Society of Anesthesiologists Physical Status of 5 or 6, temperature probes placed at sites other than the nasopharynx or the oropharynx, cases in which an endotracheal tube was not used for general anesthesia, or cases with less than 3 temperature readings in the anesthetic records. These temperature recordings were extracted from anesthesia charts. Only intraoperative readings were used for artifact detection.

After the cohort was selected by using the inclusion and exclusion criteria, a convenience sample of 200 noncardiac surgical cases from an anonymized institution within the MPOG consortium was chosen.

### End Points

The primary study end point was to assess the sensitivity and specificity of the two algorithms for detecting artifacts in automated intraoperative temperature recordings by comparing them to a reference standard—manual artifact detection by three anesthesiologists. The other study end points included measures of agreement (by case) between each algorithm and between the algorithms and the experts’ adjudications for mean temperatures and areas under the curve (AUCs). AUCs for temperature readings below 36 °C were used for this analysis. The AUC for the time multiplied by temperature readings below 36 °C was calculated for each patient after excluding artifacts, as adjudicated by the algorithms or the experts. The use of AUCs for temperature readings under 36 °C served as an index that combined the duration and severity of patient hypothermia [13].

### Algorithm Definitions

**Figure 1:**
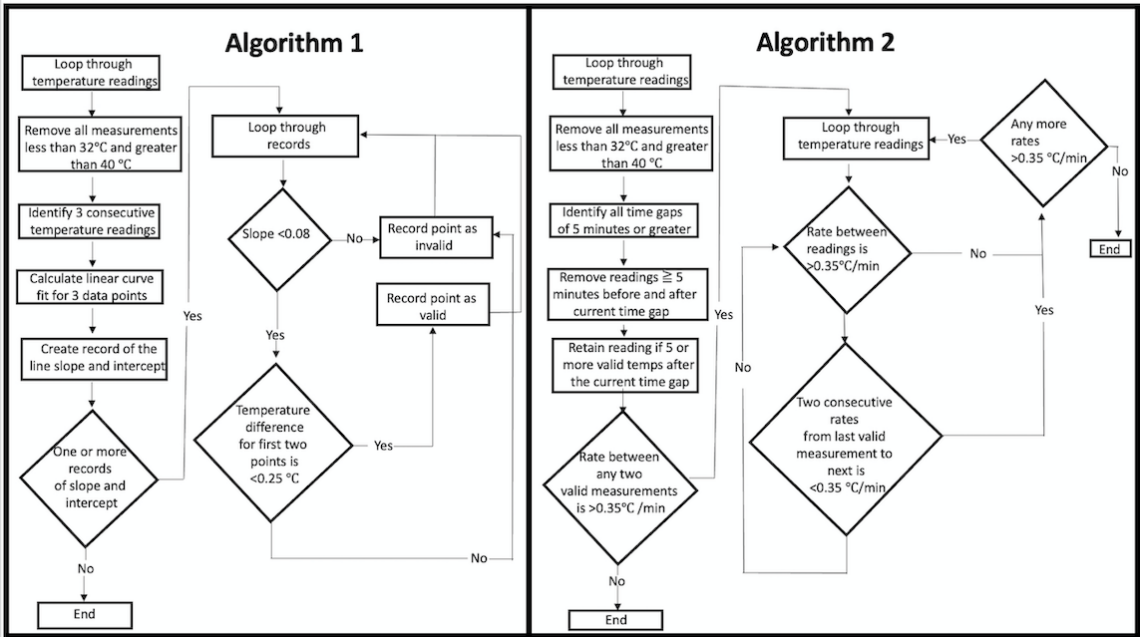
The algorithms used for the reduction of artifacts in intraoperative temperature recordings.

Algorithms 1 and 2 are depicted in Figure 1. Briefly, temperatures below 32 °C and above 40 °C were excluded. The algorithms’ logics were then used to identify potential artifacts in accordance with their flowcharts. Algorithm 1—the slope-based algorithm—calculated the linear curve fit of 3 adjacent temperature data points. Data points that had a slope of greater than 0.08 were excluded. The algorithm then calculated the absolute temperature difference between the previous data point and the next data point. Temperatures with an absolute change of greater than 0.25 °C from the previous temperature were excluded.

Algorithm 2—the interval-based algorithm—assessed for time gaps between contiguous temperature recordings that were more than 5 minutes apart. If there were less than 5 temperature recordings after the time gap, they were recorded as artifacts. If, however, there were more than 5 recordings after the measurement gap, then the slope between the last valid temperature recording and the next temperature recording was calculated, and if the slope was less than 0.35 °C per minute, then the temperature points were retained. Otherwise, they were marked as artifacts.

### Adjudication by Experts

Three board-certified anesthesiologists independently identified artifacts in temperature readings of intraoperative cases; each anesthesiologist was blinded to the other anesthesiologists’ results and the algorithms’ calculations. In the event of discordance, the majority rule (ie, agreement among at least 2 of the 3 anesthesiologists) was followed. We used an innovative approach to present time-temperature readings to the experts, for which we developed software on the JavaFX (Oracle Corporation) and Java 11 JDK (Oracle Corporation) platforms. The program first extracted patient temperature data to a flat file. Each record incorporated a unique patient identifier, temperature, and time stamp. The data were then written to an HTML file, using a FreeMarker Java template. The file used the JavaScript Google Visualization application programming interface to display intraoperative temperatures for each case in a scatterplot, which displayed temperatures on the vertical axis and time on the horizontal axis (Multimedia Appendix 1). Experts marked readings that they considered to be artifacts. The results were recorded and abstracted to a datasheet.

### Statistical Analysis

Statistical analyses were performed by using SAS version 9.4 (SAS Institute Inc). Descriptive statistics were performed on all extracted temperature readings, including readings deemed artifactual by each algorithm and the expert-adjudicated values.

The results of the manual artifact identification by the experts (majority rule) and the two algorithms were also compared by using Bland-Altman plots for both mean temperatures and AUCs for hypothermic temperature readings. For these AUCs, we computed the average height between successive time points and corresponding interval widths to estimate the segment areas. We aggregated the areas for temperatures under 36 °C to obtain the total area for each surgical case.

### Sample Size Justification

Although we conducted an observational descriptive analysis without inferential aims, we performed a power analysis to establish the extent to which the data set would define bias and limits of agreement. After a literature review, we were not able to find similar studies that could be used to guide the sample size estimation. Based on our pilot data, the mean sample difference in AUCs for temperature readings below 36 °C between the experts and each algorithm was 0.2 (SD 1.02) minutes×°C. Using the methodology developed by Lu et al [14], we determined that we would require a sample size of 147 patient records to achieve 80% power to detect agreement when the confidence level of the level of agreement was set to 0.950 and the confidence level of the CIs for the levels of agreement was set to 0.95. The maximum allowable difference was 2.56 minutes×°C, which was much lower than our prespecified, clinically meaningful value of 4 minutes×°C. To account for the possibility of including cases without any temperature recordings and to be well beyond the 80% power threshold, an a priori decision was made to include 200 intraoperative cases, which were analyzed for this study.

## Results

### Study Characteristics

A total of 27,683 temperature readings from 200 anesthetic records were analyzed by the algorithms and the anesthesiologists. The median temperature reading count per case was 103 (IQR 51-185.5). A histogram depicting the temperature curve is presented in Multimedia Appendix 2. There was unanimous agreement among the anesthesiologists for 92.1% (25,496/27,683) of the temperature readings; they identified 89 records as artifacts. An additional 200 readings were noted as artifacts by using the majority rule, resulting in a total of 289 temperature readings that were considered to be artifacts.

### Sensitivity and Specificity for Artifact Detection

Among the 27,683 temperature readings, a total of 411 temperature points were identified as artifacts by the slope-based algorithm, and 236 points were identified as artifacts by the interval-based algorithm. Notably, these rejections were not limited to a few cases. Of the 200 cases, 81 (40.5%) had at least one rejection by the slope-based algorithm, and 89 cases (44.5%) had at least one rejection by the interval-based algorithm. In comparison, 88 cases (44%) were adjudicated to have artifacts by the anesthesiologists. The mean number of rejections for each of the 200 cases was 2.1 for the slope-based algorithm and 1.2 for the interval-based algorithm.

As expected, both algorithms had a high specificity for artifact detection (slope-based algorithm: 99.02%; interval-based algorithm: 99.54%), while the slope-based algorithm appeared to be better than the interval-based algorithm in terms of sensitivity (49.13% vs 37.72%). The F-score was 0.65 for the slope-based algorithm and 0.55 for the interval-based algorithm.

### AUC Estimates for Hypothermic Temperature Readings

**Figure 2:**
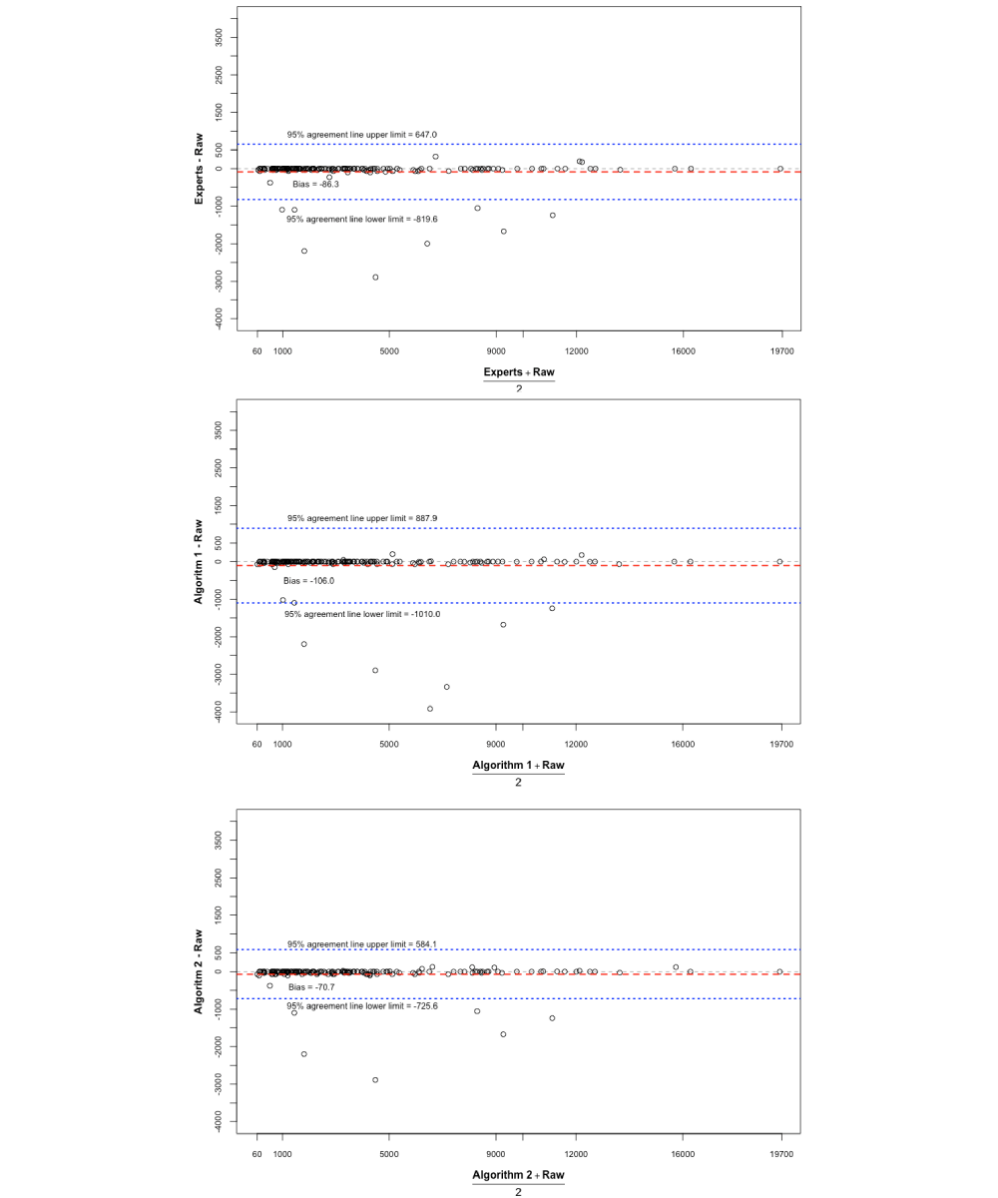
Bland-Altman plots for the interrater agreement analysis of areas under the curve for hypothermia; 95% limits of agreement are shown with light blue lines, bias is shown as a dotted black line, and the agreement bias of 2 methods is shown as a solid red line. Each dot represents a surgical case.

Comparisons between the AUCs for hypothermic temperature readings from raw data and those from anesthesiologists showed no appreciable differences in the patient-averaged summaries (Figure 2). However, some differences were seen in extremely low temperatures readings, such as the positive bias toward the raw data in the analyzed curves. This bias was seen because such low temperature readings were frequently adjudicated to be artifactual by experts and discarded in their AUC calculations, but they were used for AUC calculations with the raw data. Similar results were obtained after comparing each algorithm to the raw data (Figure 2 and Multimedia Appendix 3).

Previously, an AUC of 60 minutes×°C was used as a standard unit of reference; multiples of 60 minutes×°C were shown to be associated with adverse patient outcomes [10]. Our Bland-Altman plots indicated a bias value of greater than 60 minutes×°C between experts and raw values (−86.26 minutes×°C), between algorithm 1 and raw values (−106.04 minutes×°C), and between algorithm 2 and raw values (−70.73 minutes×°C). This indicates that the application of these algorithms may make hypothermia-based temperature analyses more meaningful than analyses based on raw data alone when assessing the impact of hypothermia on patient outcomes.

Interestingly, both the bias between experts and the slope-based algorithm (19.78 minutes×°C) and the bias between experts and the interval-based algorithm (−15.53 minutes×°C) were less than 60 minutes×°C, suggesting that after the raw data were evaluated by experts or by either of the algorithms, the resulting measures of hypothermia were similar and were within accepted measures of clinical relevance.

**Figure 3:**
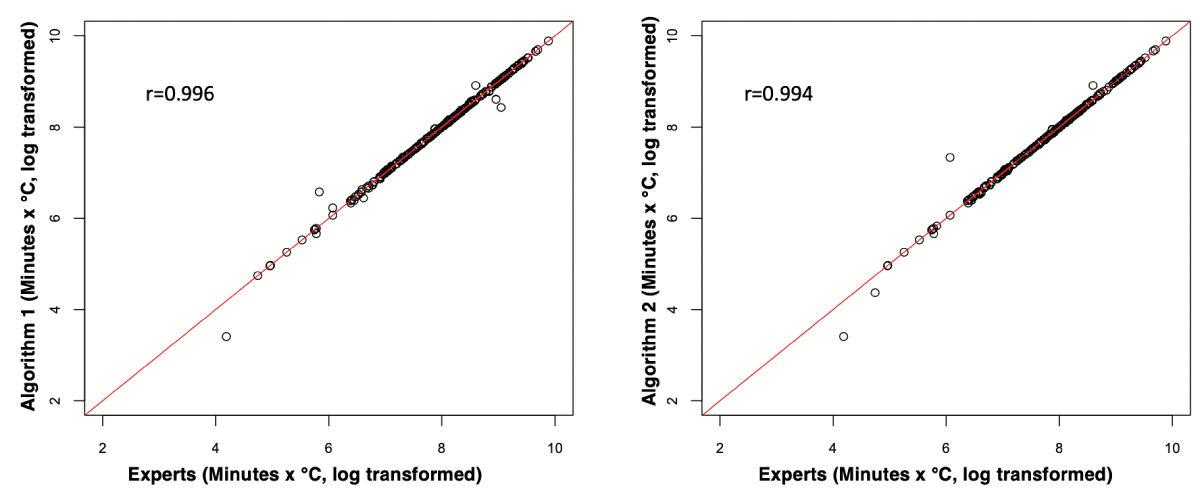
Scatter plots showing the distribution of AUCs for hypothermia (time under 36 °C × hypothermic temperature value) for the cases after artifact removal by the algorithms versus the anesthesiologists (experts). Each dot indicates a case. Values on the red line indicate cases that have temperature readings with similar AUCs after artifact removal by experts and by algorithm 1 (left) and algorithm 2 (right). Values to the right of the red line indicate fewer hypothermic temperatures marked as artifacts by the algorithm (compared to those marked by experts), leading to larger AUCs calculated by the experts compared to those calculated by the algorithms. AUC: area under the curve.

**Figure 4:**
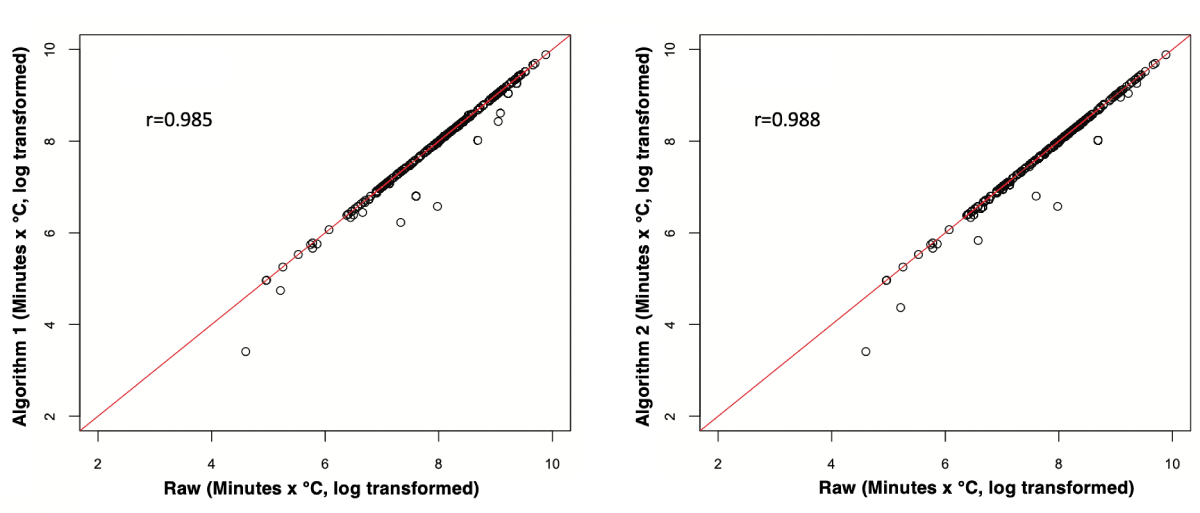
Scatter plots showing the distribution of AUCs for hypothermia (time under 36 °C × hypothermic temperature value) for the cases after artifact removal by the algorithms versus the raw values. Each dot indicates a case. Values on the red line indicate cases that have temperature readings with similar AUCs before (raw values) and after artifact removal by algorithm 1 (left) and algorithm 2 (right). Values to the right of the red line indicate the number of hypothermic temperatures marked as artifacts by the algorithm (as compared to the raw values), leading to larger AUCs calculated from the raw data compared to those calculated by the algorithms. AUC: area under the curve.

In order to better characterize the agreement, we assessed the performance of the algorithms in evaluating a clinically meaningful measure. Large AUCs for hypothermic temperature readings (time under 36 °C × temperature value of under 36 °C) have been shown to be associated with poor postoperative outcomes, including increased lengths of hospital stay and the need for a blood transfusion [10]. We used a similar approach to compare such AUCs for each case after artifact removal by experts and artifact removal by the slope-based algorithm (algorithm 1) and the interval-based algorithm (algorithm 2; Figure 3 and Figure 4). These methodologies have been used in similar studies comparing 2 modalities of measurement [15].

### Mean Temperature Estimates

**Figure 5:**
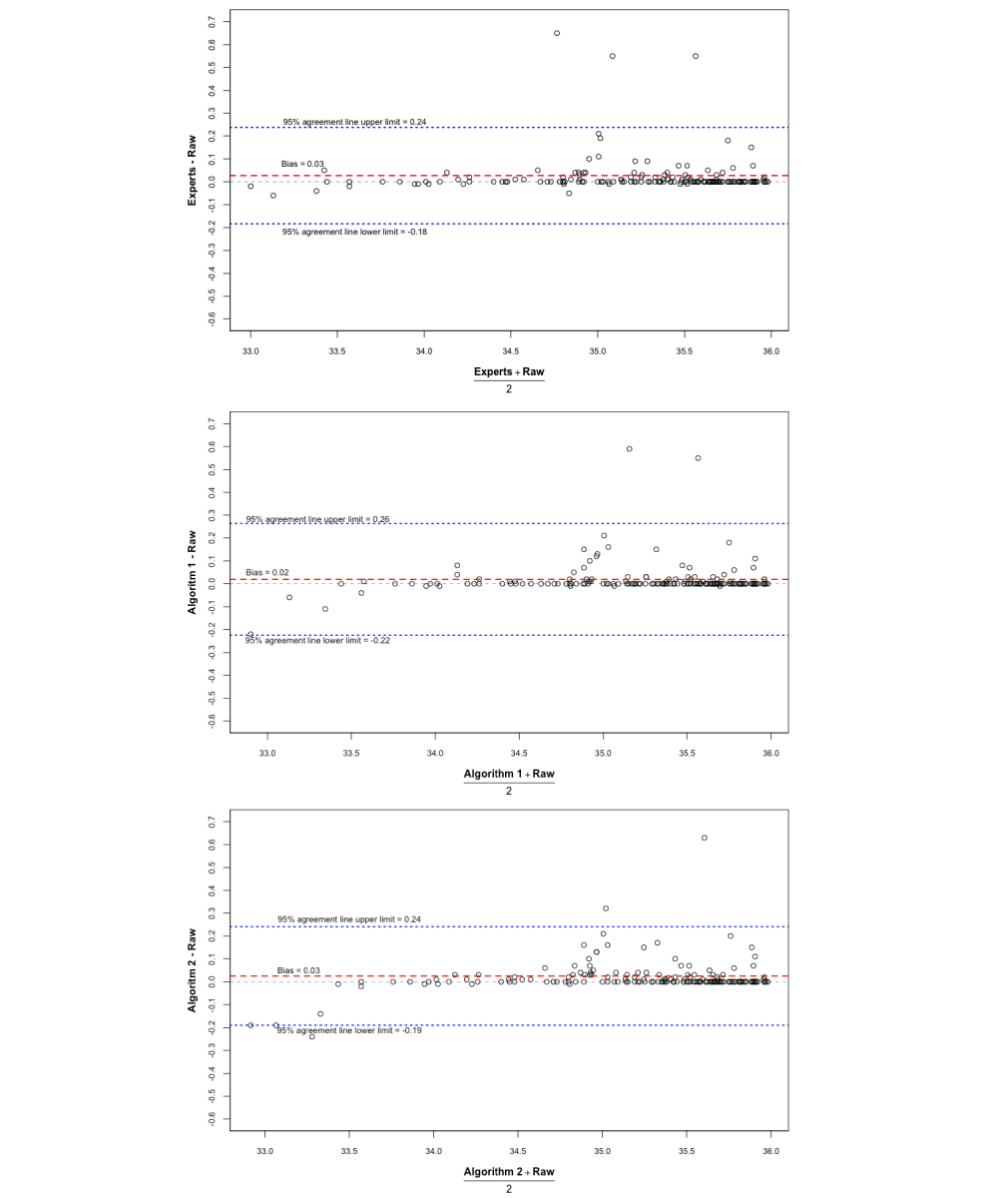
Bland-Altman plots for the interrater agreement analysis of mean temperatures; 95% limits of agreement are shown with light blue lines, bias is shown as a dotted black line, and the agreement bias of 2 methods is shown as a solid red line. Each dot represents a surgical case.

Mean temperature readings for each patient record were calculated after artifact removal via the methods we described. The mean temperature reading profiles, in which the raw data were compared to anesthesiologists’ majority rule–based results, showed no appreciable differences (Figure 5). However, as with the AUCs, bias in mean temperature was seen at extremely low temperatures. The mean temperature readings for each of the two algorithms before and after artifact removal followed a similar trend (Figure 5 and Multimedia Appendix 4).

### Clustering of the Artifacts

In order to describe clusters, we considered a cluster to be 3 or more consecutive temperature readings that were adjudicated as artifacts. We compared the distributions of the number of clusters per case among the three methods (manual artifact detection by anesthesiologists, the use of the slope-based algorithm [algorithm 1], and the use of the interval-based algorithm [algorithm 2]), as depicted in Multimedia Appendix 5. There was very good interrater reliability for the number of artifactual data points (Gwet AC1 statistic 0.876, 95% CI 0.833-0.92) [16]. The distributions of the cluster sizes in each case among the three methods is shown in Multimedia Appendix 6.

## Discussion

This study has important findings. First, the overall rate of intraoperative temperature artifacts in the sample, which was obtained via automated electronic health record data capture, was low (point estimate 0.01, 95% CI 0.009-0.011). To the best of our knowledge, our study is the first of its kind to address the validity of raw intraoperative temperature recordings. Thus, mean temperature values derived from raw data closely approximate those derived by experts and may be directly used for research purposes. Second, the slope-based algorithm can filter intraoperative temperature artifacts, closely approximating manual sorting by anesthesiologists. The artifact reduction algorithm can thus be used by studies that evaluate the effect of intraoperative hypothermia on patient outcomes. This algorithm can also serve as a powerful tool for gauging the quality of temperature data capture by a particular medical center via comparisons to other medical centers. In addition, our methodology can be used to validate similar algorithms aimed at discerning artifacts associated with other vitals, such as intraoperative blood pressure.

Our intraoperative temperature recordings are similar to those in other studies evaluating intraoperative temperatures [17,18]. The majority of patients under general anesthesia tend to experience a decrease in core body temperature [19,20]. This pattern of change varies widely based on the type and duration of surgery [21]. We saw similar patterns in our random sample of intraoperative temperature records, which indicated that our sample was not biased toward a particular subset of patients or surgeries. Studies that have attempted to filter out artifacts related to intraoperative temperature measurements lack generalizability [10]. One of the key strengths of our study is that, given the adaptability of the algorithms, they can be applied by a particular medical center to filter intraoperative artifacts both for research and for quality initiative purposes.

Our study has some limitations. First, due to the lack of a true gold standard, manual artifact sorting by anesthesiologists was considered a reasonable method for assessing the performance of artifact detection. An alternate methodology for measuring the artifacts could have been correlating esophageal temperatures with temperature measurements that were simultaneously captured from other sites, such as the bladder. However, very few patients receive more than 1 temperature measurement modality. Moreover, bladder temperatures lag behind esophageal temperatures, which would make identifying a true artifact difficult [1]. Additionally, each modality has its own limitations, undermining the very notion of any single gold standard source of core temperature readings. For example, bladder temperature measurement devices are strongly influenced by urine flow [22]. Second, the algorithms were only validated for cases in which nasopharyngeal or oropharyngeal temperature probes were used. However, these probes are used for intraoperative temperature measurement among the vast majority of patients. Third, this study had a retrospective design. We chose to use this study design, as conducting this study in real time would have been very resource intensive. Moreover, having an observer could have resulted in changes in clinician behavior. Due to the retrospective nature of this study, we included cases in which patient temperature data were automatically acquired via oropharyngeal probes or nasopharyngeal probes. These probes generally use thermistors or thermocouples, which are considered standard for clinical use, though this was not a standardized part of the study protocol, given its retrospective design [1]. Further, the algorithms’ calculations are based on changes in temperature and gradients of temperature change during an anesthetic encounter, and the algorithms would fail to detect artifacts if temperature probes were inappropriately placed for the entirety of an anesthetic encounter. Fourth, it is possible that outliers could have heavily influenced our observations. In order to address this, we performed sensitivity analyses of our results by re-estimating our result summaries via the jackknife method—a “leave one observation out at a time” approach. The jackknife estimates revealed that there were no highly influential observations. The bias estimates remained largely unchanged (Multimedia Appendix 7). Fifth, we would also like to emphasize that while the slope-based algorithm achieved a modest F-score, significant room for improvement exists. However, this algorithm may be an important first step in addressing the validity of automated intraoperative temperature recordings and may serve as a scaffold for further improved algorithms. Finally, the algorithms may poorly extrapolate temperature-time curves that include time gaps or time periods in which data were not collected, and they may mask intraoperative temperature shifts that could have occurred during these periods. Such anomalies, however, happened infrequently and were detectable upon investigation.

In summary, it is widely recognized that intraoperative temperature monitoring is key to postoperative patient outcomes. Our study provides highly generalizable artifact reduction algorithms that can be used as standard open-access tools to filter out artifacts in large database studies. They can also be used as tools for assessing the quality of intraoperative temperature recordings at various centers. Further investigations should assess our slope-based algorithm’s performance for other intraoperative databases and populations.

## Appendix

Multimedia Appendix 1 Intraoperative temperatures for a surgical case displayed as a function of time. Red dots indicate the temperature points that were adjudicated by an anesthesiologist as artifactual.

Multimedia Appendix 2 Histogram showing the distribution of raw temperature data in the study cohort.

Multimedia Appendix 3 Bland-Altman plots for the interrater agreement analysis of areas under the curve (AUCs) for hypothermia; 95% limits of agreement are shown with light blue lines, bias is shown as a dotted black line, and the agreement bias of 2 methods is shown as a solid red line. Each dot represents a surgical case.

Multimedia Appendix 4 Bland-Altman plots for the interrater agreement analysis of mean temperature; 95% limits of agreement are shown with light blue lines, bias is shown as a dotted black line, and the agreement bias of 2 methods is shown as a solid red line. Each dot represents a surgical case.

Multimedia Appendix 5 Distribution of the number of temperature clusters (3 consecutive artifactual temperature readings), as adjudicated by experts, algorithm 1, and algorithm 2.

Multimedia Appendix 6 Distribution of the size of the clusters per case for the three methods (experts, algorithm 1, and algorithm 2).

Multimedia Appendix 7 Table depicting jackknife analysis versus full data to understand potential outlier effects.

## Abbreviations

AUC: area under the curve
MPOG: Multicenter Perioperative Outcomes Group

## References

[1] Sessler DI (2008). Temperature monitoring and perioperative thermoregulation. Anesthesiology.

[2] Standards for basic anesthetic monitoring. American Society of Anesthesiologists.

[3] Sessler DI (2001). Complications and treatment of mild hypothermia. Anesthesiology.

[4] Lenhardt R, Marker E, Goll V, Tschernich H, Kurz A, Sessler DI, Narzt E, Lackner F (1997). Mild intraoperative hypothermia prolongs postanesthetic recovery. Anesthesiology.

[5] Kurz A, Sessler DI, Lenhardt R (1996). Perioperative normothermia to reduce the incidence of surgical-wound infection and shorten hospitalization. Study of Wound Infection and Temperature Group. N Engl J Med.

[6] Frank SM, Fleisher LA, Breslow MJ, Higgins MS, Olson KF, Kelly S, Beattie C (1997). Perioperative maintenance of normothermia reduces the incidence of morbid cardiac events. A randomized clinical trial. JAMA.

[7] Berríos-Torres SI, Umscheid CA, Bratzler DW, Leas B, Stone EC, Kelz RR, Reinke CE, Morgan S, Solomkin JS, Mazuski JE, Dellinger EP, Itani KMF, Berbari EF, Segreti J, Parvizi J, Blanchard J, Allen G, Kluytmans JAJW, Donlan R, Schecter WP, Healthcare Infection Control Practices Advisory Committee (2017). Centers for Disease Control and Prevention guideline for the prevention of surgical site infection, 2017. JAMA Surg.

[8] Lee J, Lim H, Son KG, Ko S (2014). Optimal nasopharyngeal temperature probe placement. Anesth Analg.

[9] Wang M, Singh A, Qureshi H, Leone A, Mascha EJ, Sessler DI (2016). Optimal depth for nasopharyngeal temperature probe positioning. Anesth Analg.

[10] Sun Z, Honar H, Sessler DI, Dalton JE, Yang D, Panjasawatwong K, Deroee AF, Salmasi V, Saager L, Kurz A (2015). Intraoperative core temperature patterns, transfusion requirement, and hospital duration in patients warmed with forced air. Anesthesiology.

[11] Kheterpal S, Vaughn MT, Dubovoy TZ, Shah NJ, Bash LD, Colquhoun DA, Shanks AM, Mathis MR, Soto RG, Bardia A, Bartels K, McCormick PJ, Schonberger RB, Saager L (2020). Sugammadex versus neostigmine for reversal of neuromuscular blockade and postoperative pulmonary complications (STRONGER): A multicenter matched cohort analysis. Anesthesiology.

[12] Schonberger R (2020). Demonstration and performance evaluation of two novel algorithms to remove artifacts from automated intraoperative temperature datasets. Center for Open Science.

[13] Hopf HW (2015). Perioperative temperature management: time for a new standard of care?. Anesthesiology.

[14] Lu MJ, Zhong WH, Liu YX, Miao HZ, Li YC, Ji MH (2016). Sample size for assessing agreement between two methods of measurement by Bland-Altman method. Int J Biostat.

[15] Schumann R, Meidert AS, Bonney I, Koutentis C, Wesselink W, Kouz K, Saugel B (2021). Intraoperative blood pressure monitoring in obese patients. Anesthesiology.

[16] Gwet K (2002). Inter-rater reliability: Dependency on trait prevalence and marginal homogeneity. Statistical Methods For Inter-Rater Reliability Assessment.

[17] Yi J, Xiang Z, Deng X, Fan T, Fu R, Geng W, Guo R, He N, Li C, Li L, Li M, Li T, Tian M, Wang G, Wang L, Wang T, Wu A, Wu D, Xue X, Xu M, Yang X, Yang Z, Yuan J, Zhao Q, Zhou G, Zuo M, Pan S, Zhan L, Yao M, Huang Y (2015). Incidence of inadvertent intraoperative hypothermia and its risk factors in patients undergoing general anesthesia in Beijing: A prospective regional survey. PLoS One.

[18] Seamon MJ, Wobb J, Gaughan JP, Kulp H, Kamel I, Dempsey DT (2012). The effects of intraoperative hypothermia on surgical site infection: an analysis of 524 trauma laparotomies. Ann Surg.

[19] John M, Crook D, Dasari K, Eljelani F, El-Haboby A, Harper CM (2016). Comparison of resistive heating and forced-air warming to prevent inadvertent perioperative hypothermia. Br J Anaesth.

[20] Andrzejowski J, Hoyle J, Eapen G, Turnbull D (2008). Effect of prewarming on post-induction core temperature and the incidence of inadvertent perioperative hypothermia in patients undergoing general anaesthesia. Br J Anaesth.

[21] Kasai T, Hirose M, Yaegashi K, Matsukawa T, Takamata A, Tanaka Y (2002). Preoperative risk factors of intraoperative hypothermia in major surgery under general anesthesia. Anesth Analg.

[22] Horrow JC, Rosenberg H (1988). Does urinary catheter temperature reflect core temperature during cardiac surgery?. Anesthesiology.

