# Supplementary figures and images for: Demonstration and Performance Evaluation of Two Novel Algorithms for Removing Artifacts From Automated Intraoperative Temperature Data Sets: Multicenter, Observational, Retrospective Study
Source: JMIR Perioper Med. 2022 Oct 5;5(1):e37174. doi: 10.2196/37174 (PMC9591708; doi:10.2196/37174)

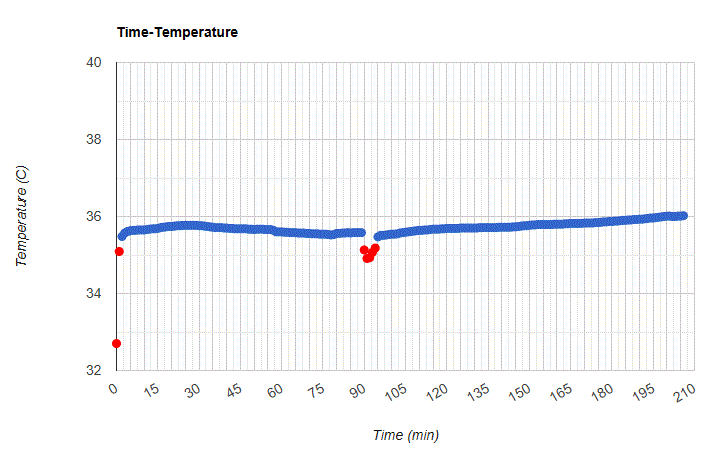

Supplement: Multimedia Appendix 1 [file periop_v5i1e37174_app1.png]

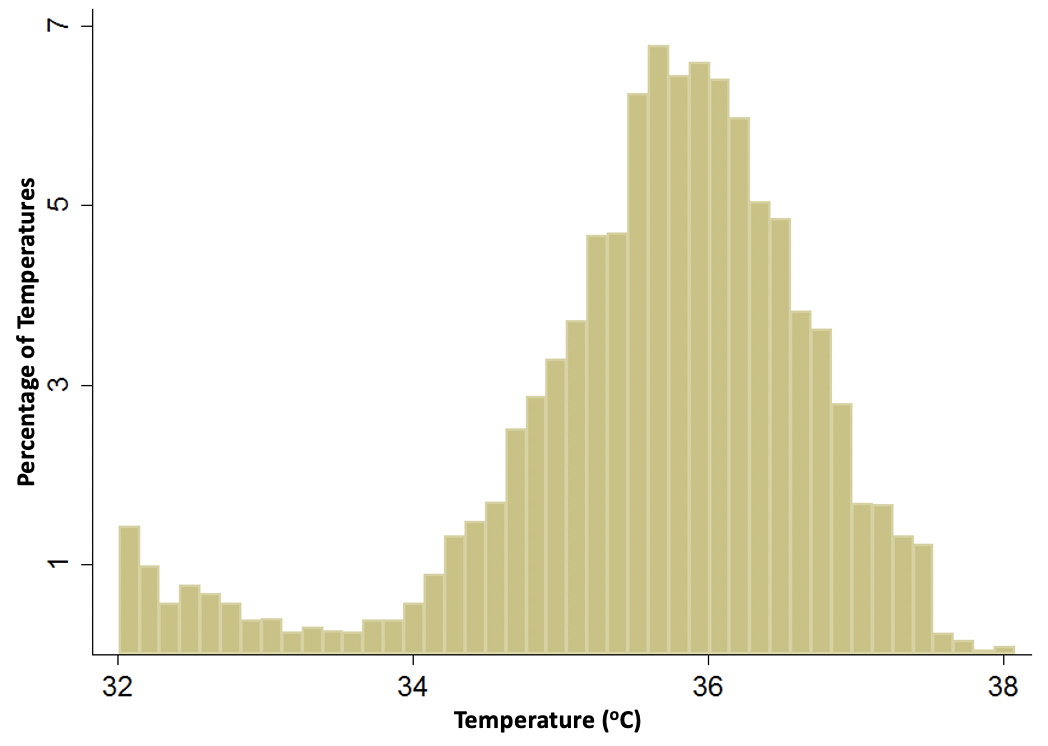

Supplement: Multimedia Appendix 2 [file periop_v5i1e37174_app2.png]

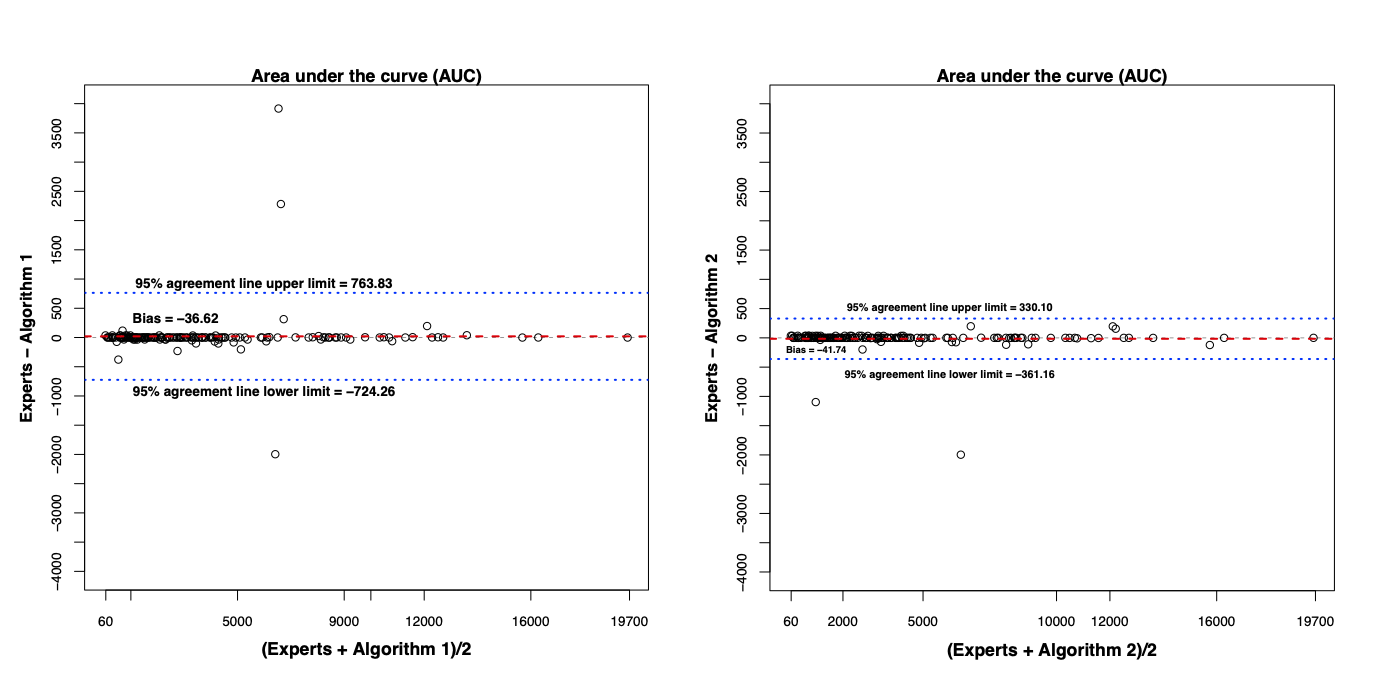

Supplement: Multimedia Appendix 3 [file periop_v5i1e37174_app3.png]

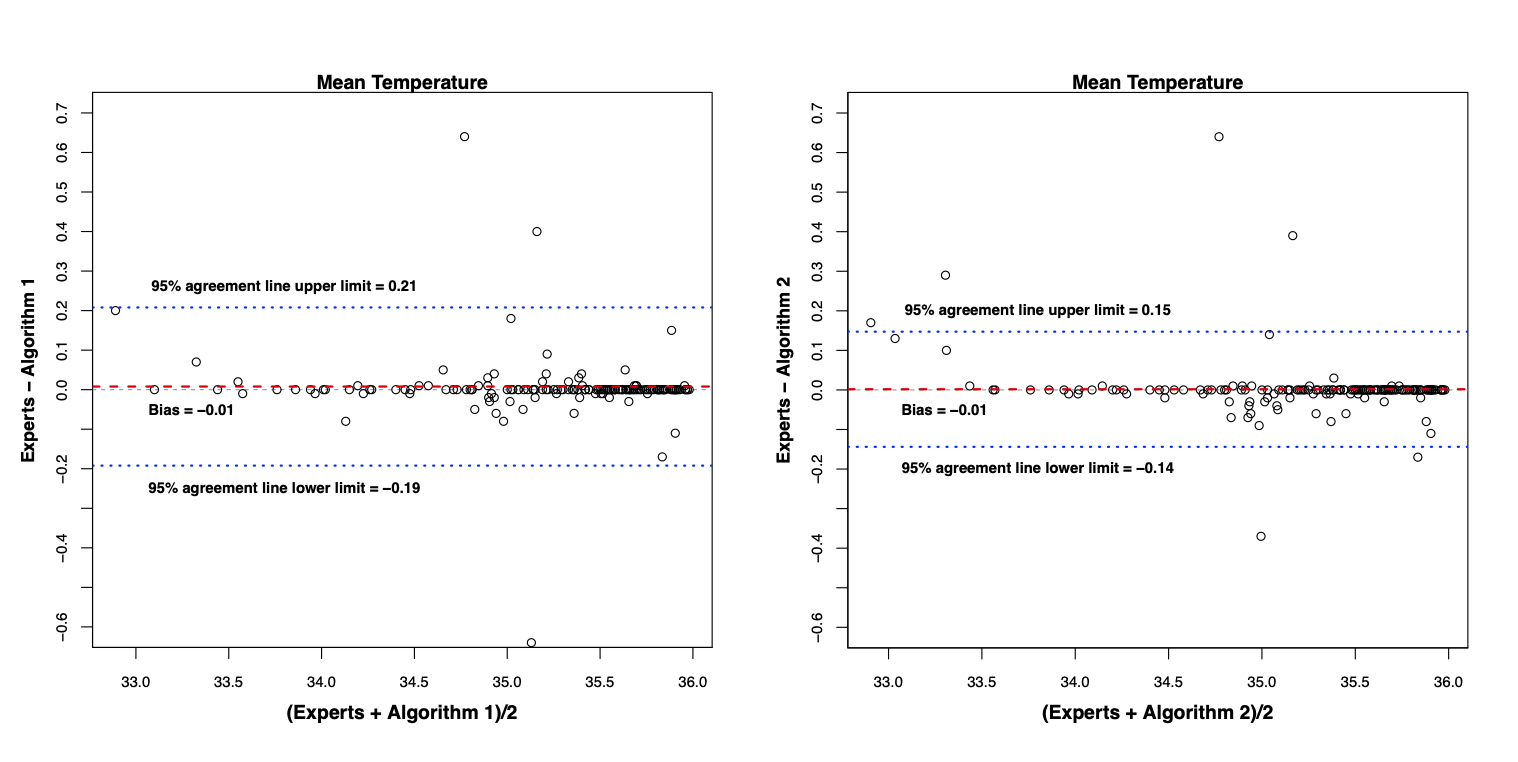

Supplement: Multimedia Appendix 4 [file periop_v5i1e37174_app4.png]

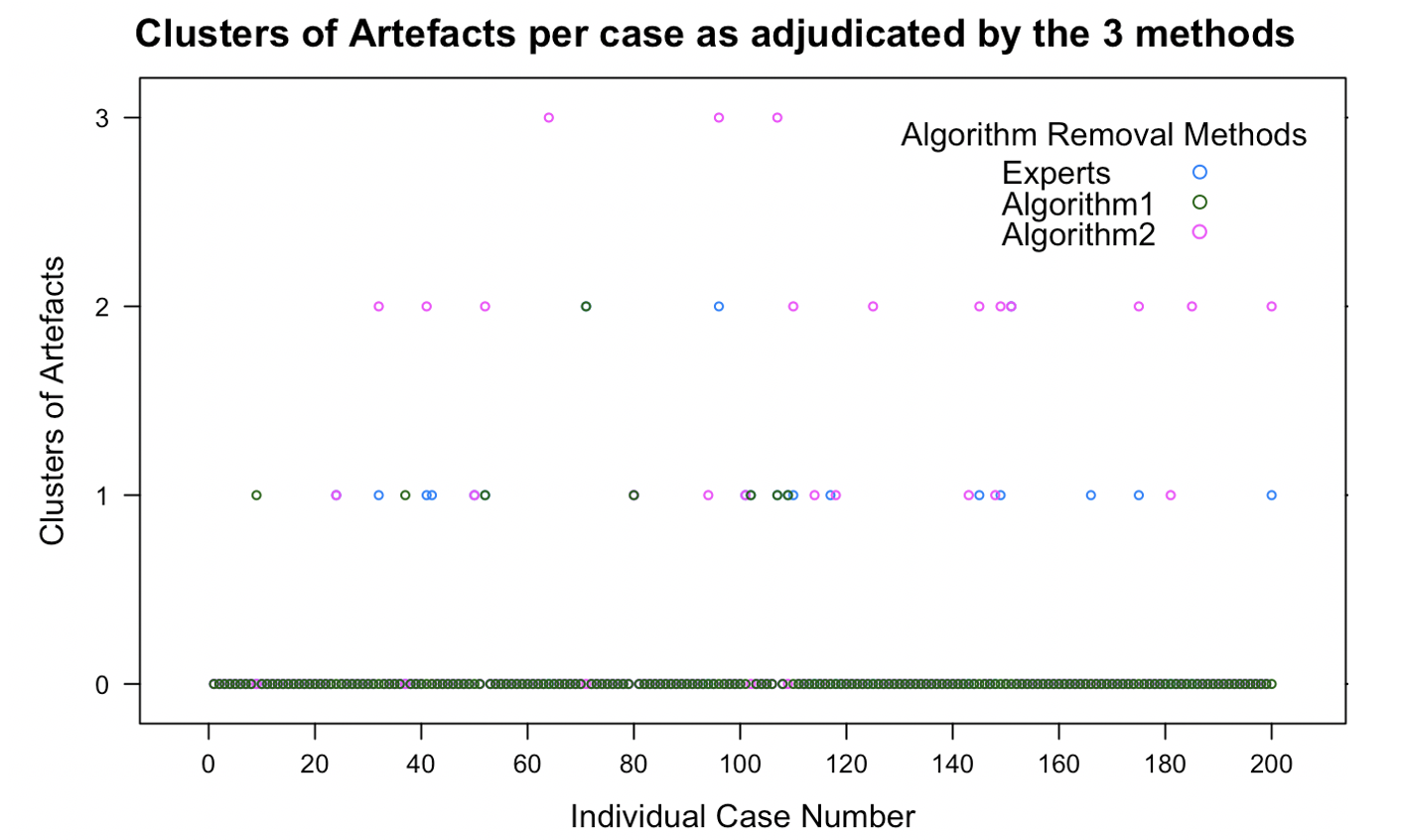

Supplement: Multimedia Appendix 5 [file periop_v5i1e37174_app5.png]
