# Supplementary material for: Demonstration and Performance Evaluation of Two Novel Algorithms for Removing Artifacts From Automated Intraoperative Temperature Data Sets: Multicenter, Observational, Retrospective Study
Source: JMIR Perioper Med. 2022 Oct 5;5(1):e37174. doi: 10.2196/37174 (PMC9591708; doi:10.2196/37174)
Supplement: Multimedia Appendix 6 [file periop_v5i1e37174_app6.docx]

**Supplement Table 1:** Distribution of size of the clusters per case by the three methods (Experts, Algorithm 1 and Algorithm 2).

| Case Number | Artefact cluster size as adjudicated by  Experts, Algorithm 1 and Algorithm 2 |
| --- | --- |
| 1 | [[0], [0], [0]] |
| 2 | [[0], [0], [0]] |
| 3 | [[0], [0], [0]] |
| 4 | [[0], [0], [0]] |
| 5 | [[0], [0], [0]] |
| 6 | [[0], [0], [0]] |
| 7 | [[0], [0], [0]] |
| 8 | [[0], [0], [0]] |
| 9 | [[0], [0], [3]] |
| 10 | [[0], [0], [0]] |
| 11 | [[0], [0], [0]] |
| 12 | [[0], [0], [0]] |
| 13 | [[0], [0], [0]] |
| 14 | [[0], [0], [0]] |
| 15 | [[0], [0], [0]] |
| 16 | [[0], [0], [0]] |
| 17 | [[0], [0], [0]] |
| 18 | [[0], [0], [0]] |
| 19 | [[0], [0], [0]] |
| 20 | [[0], [0], [0]] |
| 21 | [[0], [0], [0]] |
| 22 | [[0], [0], [0]] |
| 23 | [[0], [0], [0]] |
| 24 | [[4], [12], [3, 8]] |
| 25 | [[0], [0], [0]] |
| 26 | [[0], [0], [0]] |
| 27 | [[0], [0], [0]] |
| 28 | [[0], [0], [0]] |
| 29 | [[0], [0], [0]] |
| 30 | [[0], [0], [0]] |
| 31 | [[0], [0], [0]] |
| 32 | [[5], [5, 9], [6]] |
| 33 | [[0], [0], [0]] |
| 34 | [[0], [0], [0]] |
| 35 | [[0], [0], [0]] |
| 36 | [[0], [0], [0]] |
| 37 | [[0], [0], [3]] |
| 38 | [[0], [0], [0]] |
| 39 | [[0], [0], [0]] |
| 40 | [[0], [0], [0]] |
| 41 | [[5], [4, 14], [0]] |
| 42 | [[3], [0], [3]] |
| 43 | [[0], [0], [0]] |
| 44 | [[0], [0], [0]] |
| 45 | [[0], [0], [0]] |
| 46 | [[0], [0], [0]] |
| 47 | [[0], [0], [0]] |
| 48 | [[0], [0], [0]] |
| 49 | [[0], [0], [0]] |
| 50 | [[3], [10], [7]] |
| 51 | [[0], [0], [0]] |
| 52 | [[75], [5, 9], [4, 8]] |
| 53 | [[0], [0], [0]] |
| 54 | [[0], [0], [0]] |
| 55 | [[0], [0], [0]] |
| 56 | [[0], [0], [0]] |
| 57 | [[0], [0], [0]] |
| 58 | [[0], [0], [0]] |
| 59 | [[0], [0], [0]] |
| 60 | [[0], [0], [0]] |
| 61 | [[0], [0], [0]] |
| 62 | [[0], [0], [4]] |
| 63 | [[0], [0], [0]] |
| 64 | [[0], [6, 7, 3], [0]] |
| 65 | [[0], [0], [0]] |
| 66 | [[0], [0], [0]] |
| 67 | [[0], [0], [3]] |
| 68 | [[0], [0], [0]] |
| 69 | [[0], [0], [0]] |
| 70 | [[0], [0], [0]] |
| 71 | [[3, 3], [0], [0]] |
| 72 | [[0], [0], [0]] |
| 73 | [[0], [0], [0]] |
| 74 | [[0], [0], [0]] |
| 75 | [[0], [0], [0]] |
| 76 | [[0], [0], [0]] |
| 77 | [[0], [0], [0]] |
| 78 | [[0], [0], [0]] |
| 79 | [[0], [0], [0]] |
| 80 | [[3], [11], [0]] |
| 81 | [[0], [0], [0]] |
| 82 | [[0], [0], [0]] |
| 83 | [[0], [0], [0]] |
| 84 | [[0], [0], [0]] |
| 85 | [[0], [0], [0]] |
| 86 | [[0], [0], [0]] |
| 87 | [[0], [0], [0]] |
| 88 | [[0], [0], [0]] |
| 89 | [[0], [0], [0]] |
| 90 | [[0], [0], [0]] |
| 91 | [[0], [0], [0]] |
| 92 | [[0], [0], [0]] |
| 93 | [[0], [0], [0]] |
| 94 | [[0], [3], [4]] |
| 95 | [[0], [0], [0]] |
| 96 | [[4, 3], [4, 6, 14], [0]] |
| 97 | [[0], [0], [0]] |
| 98 | [[0], [0], [0]] |
| 99 | [[0], [0], [0]] |
| 100 | [[0], [0], [0]] |
| 101 | [[3], [6], [0]] |
| 102 | [[5], [0], [0]] |
| 103 | [[0], [0], [0]] |
| 104 | [[0], [0], [0]] |
| 105 | [[0], [0], [0]] |
| 106 | [[0], [0], [0]] |
| 107 | [[3], [4, 3, 3], [3]] |
| 108 | [[0], [0], [0]] |
| 109 | [[7], [0], [0]] |
| 110 | [[5], [4, 12], [3]] |
| 111 | [[0], [0], [0]] |
| 112 | [[0], [0], [0]] |
| 113 | [[0], [0], [0]] |
| 114 | [[0], [8], [0]] |
| 115 | [[0], [0], [0]] |
| 116 | [[0], [0], [0]] |
| 117 | [[3], [0], [3]] |
| 118 | [[0], [3], [0]] |
| 119 | [[0], [0], [0]] |
| 120 | [[0], [0], [0]] |
| 121 | [[0], [0], [0]] |
| 122 | [[0], [0], [0]] |
| 123 | [[0], [0], [0]] |
| 124 | [[0], [0], [0]] |
| 125 | [[0], [4, 10], [3, 8]] |
| 126 | [[0], [0], [0]] |
| 127 | [[0], [0], [0]] |
| 128 | [[0], [0], [0]] |
| 129 | [[0], [0], [0]] |
| 130 | [[0], [0], [0]] |
| 131 | [[0], [0], [0]] |
| 132 | [[0], [0], [0]] |
| 133 | [[0], [0], [0]] |
| 134 | [[0], [0], [0]] |
| 135 | [[0], [0], [0]] |
| 136 | [[0], [0], [0]] |
| 137 | [[0], [0], [0]] |
| 138 | [[0], [0], [0]] |
| 139 | [[0], [0], [0]] |
| 140 | [[0], [0], [0]] |
| 141 | [[0], [0], [0]] |
| 142 | [[0], [0], [0]] |
| 143 | [[0], [12], [0]] |
| 144 | [[0], [0], [0]] |
| 145 | [[4], [3, 14], [0]] |
| 146 | [[0], [0], [0]] |
| 147 | [[0], [0], [0]] |
| 148 | [[0], [3], [3]] |
| 149 | [[4], [3, 11], [3]] |
| 150 | [[0], [0], [0]] |
| 151 | [[5, 3], [4, 11], [3, 7]] |
| 152 | [[0], [0], [0]] |
| 153 | [[0], [0], [0]] |
| 154 | [[0], [0], [0]] |
| 155 | [[0], [0], [0]] |
| 156 | [[0], [0], [0]] |
| 157 | [[0], [0], [0]] |
| 158 | [[0], [0], [0]] |
| 159 | [[0], [0], [0]] |
| 160 | [[0], [0], [0]] |
| 161 | [[0], [0], [0]] |
| 162 | [[0], [0], [0]] |
| 163 | [[0], [0], [0]] |
| 164 | [[0], [0], [0]] |
| 165 | [[0], [0], [0]] |
| 166 | [[3], [0], [0]] |
| 167 | [[0], [0], [0]] |
| 168 | [[0], [0], [0]] |
| 169 | [[0], [0], [0]] |
| 170 | [[0], [0], [4]] |
| 171 | [[0], [0], [0]] |
| 172 | [[0], [0], [3]] |
| 173 | [[0], [0], [0]] |
| 174 | [[0], [0], [0]] |
| 175 | [[5], [5, 4], [4]] |
| 176 | [[0], [0], [0]] |
| 177 | [[0], [0], [0]] |
| 178 | [[0], [0], [0]] |
| 179 | [[0], [0], [0]] |
| 180 | [[0], [0], [0]] |
| 181 | [[0], [8], [0]] |
| 182 | [[0], [0], [0]] |
| 183 | [[0], [0], [0]] |
| 184 | [[0], [0], [0]] |
| 185 | [[0], [5, 12], [0]] |
| 186 | [[0], [0], [0]] |
| 187 | [[0], [0], [0]] |
| 188 | [[0], [0], [0]] |
| 189 | [[0], [0], [0]] |
| 190 | [[0], [0], [0]] |
| 191 | [[0], [0], [0]] |
| 192 | [[0], [0], [0]] |
| 193 | [[0], [0], [0]] |
| 194 | [[0], [0], [0]] |
| 195 | [[0], [0], [0]] |
| 196 | [[0], [0], [0]] |
| 197 | [[0], [0], [0]] |
| 198 | [[0], [0], [0]] |
| 199 | [[0], [0], [0]] |
| 200 | [[18], [7, 10], [0]] |
