# Supplementary material for: Demonstration and Performance Evaluation of Two Novel Algorithms for Removing Artifacts From Automated Intraoperative Temperature Data Sets: Multicenter, Observational, Retrospective Study
Source: JMIR Perioper Med. 2022 Oct 5;5(1):e37174. doi: 10.2196/37174 (PMC9591708; doi:10.2196/37174)
Supplement: Multimedia Appendix 7 [file periop_v5i1e37174_app7.docx]

Sensitivity analyses of our results was conducted by re-estimating our results summaries using a Jackknife analysis (i.e. a "leave one observation out at a time" approach. The Jackknife estimates reveal highly similar results to the full data analysis as outlined below (i.e. the bias estimates remain largely unchanged). The results are shown in the table below:

**Jackknife Analysis vs. Full Data to Understand Potential Outlier Effects**

| **Hypothermic Area Under Curve analysis** | **Full Data Mean Bias** | **Jackknife Data Mean Difference** | **SD of Jackknife Calculated Mean Bias** |
| --- | --- | --- | --- |
|  |  |  |  |
| Experts vs. Raw | -86.28 | -86.26 | 2.16 |
| Algorithm 1 vs. Raw | -106.04 | -106.04 | 2.93 |
| Algorithm 2 vs. Raw | -70.73 | -70.73 | 1.93 |
| Experts vs. Algorithm 1 | 19.78 | 19.78 | 2.19 |
| Experts vs. Algorithm 2 | -15.53 | -15.53 | 1.02 |
